# Responsiveness and minimal important differences after revision total hip arthroplasty

**DOI:** 10.1186/1471-2474-11-261

**Published:** 2010-11-12

**Authors:** Hon-Yi Shi, Je-Ken Chang, Chi-Yin Wong, Jun-Wen Wang, Yuan-Kun Tu, Herng-Chia Chiu, King-Teh Lee

**Affiliations:** 1 Graduate Institute of Healthcare Administration, Kaohsiung Medical University, 100, Shih-Chun 1st Road, Kaohsiung, Taiwan; 2 Department of Orthopaedics, Kaohsiung Medical University Hospital 100, Tzyou 1st Road, Kaohsiung, Taiwan; 3 Department of Orthopaedics, Kaohsiung Veterans General Hospital 386, Ta-Chung 1st Road, Kaohsiung, Taiwan; 4 Department of Orthopaedics, Chang-Gung Memorial Hospital, Kaohsiung Branch 123, Ta-Pei Road, Niao-Sung Hsiang, Kaohsiung, Taiwan; 5 Department of Orthopaedics, E-Da Hospital 1, Yi-Da Road, Jiau-Shu Shiang, Kaohsiung, Taiwan

## Abstract

**Background:**

The health-related quality of life (HRQoL) is currently weighted more heavily when evaluating health status, particularly regarding medical treatments and interventions. However, it is rarely used by physicians to compare responsiveness. Additionally, responsiveness estimates derived by the Harris Hip Score (HHS) and the Short Form 36 (SF-36) before and after revision total hip arthroplasty (THA) have not been clinically compared. This study compared responsiveness and minimal important differences (MID) between HHS and SF-36.

**Methods:**

All revision THA patients completed the disease-specific HHS and the generic SF-36 before and 6 months after surgery. Scores using these instruments were interpreted by generalized estimating equation (GEE) before and after revision THA. The bootstrap estimation and modified Jacknife test were used to derive 95% confidence intervals for differences in the responsiveness estimates.

**Results:**

Comparisons of effect size (ES), standardized response means (SRM), relative efficiency (RE) (>1) and MID indicated that the responsiveness of HHS was superior to that of SF-36. The ES and SRM for pain and physical functions in the HHS were significantly larger than those of the SF-36 (p < 0.001).

**Conclusion:**

The data in this study indicated that clinicians and health researchers should weight disease-specific measures more heavily than generic measures when evaluating treatment outcomes.

## Background

Pain and physical function outcomes of total hip arthroplasty (THA) have been well documented during the past twenty years [1,2]. This intervention has proven safe and effective for improving health-related quality of life (HRQoL) [3].

Various HRQoL instruments have been used with increasing frequency during the past decade [3]. Disease-specific measures are traditionally administered in longitudinal studies to detect progressive changes in health and quality of life after interventions and tend to focus on physical function and pain. Conversely, generic measures are designed to assess the effects of any disease or condition and have value for measuring health status. The Harris Hip Score (HHS) is a commonly used physician assessment of physical functioning and pain relief on clinical sites [4]. The Medical Outcomes Study Short Form-36 Health Survey (SF-36) is a self-administered generic HRQoL instrument commonly used to assess overall outcome [5].

Responsiveness is measured by comparing changes in clinical endpoints and changes in instrument outcomes over time in either observational or clinical trials [6,7]. Responsiveness is an important consideration when selecting HRQoL measures for clinical trials or medical interventions. Minimal important difference (MID) is defined as the smallest change in a score for a patient that indicates an actual change between two time points; that is, the MID is the minimum change in a score that likely reflects actual change rather than a variation in measurement [8,9].

The HRQoL is currently weighted more heavily when evaluating health status, particularly regarding medical treatments and interventions. Nevertheless, it is easy to identify the statistical significance of any such changes, but it can be harder to determine whether these changes are clinically or not clinically important. The MID is a statistical value arising from the variance in measure [8]. However, it is rarely used by physicians to compare responsiveness. Additionally, responsiveness estimates derived by the SF-36 and the HHS before and after revision THA has not been clinically compared.

In this prospective cohort study, two well-known HRQoL instruments, the SF-36 and the HHS, were used to compare responsiveness and MID in revision THA patients.

## Methods

### Patients and data collection

Two HRQoL instruments were used to survey all patients who underwent revision THA performed by either of two experienced surgeons practicing at two academic hospitals in southern Taiwan between October, 2007 and December, 2008. Eight procedures performed by other low-volume surgeons who had performed less than three procedures annually were excluded from analysis. Patients with cognitive impairment, severe organ or psychiatric diseases (n = 5) were excluded. Of the seventy-two eligible subjects who gave written consent and were enrolled in the study at baseline, five were excluded because they did not undergo postoperative assessments. Sixty-seven patients who completed preoperative and 6-month surveys after revision THA were enrolled in the study. Immediately before surgery, the two operating surgeons administered the HHS and a trained research assistant administered the SF-36 Health Survey. The same orthopaedists and research assistant continued to use these instruments to assess HRQoL in the 6-month survey.

### Outcome measures

The two HRQoL survey instruments in this study were the generic Chinese version of the SF-36 and the Harris Hip Score. The SF-36 Health Survey, a widely used measure of generic HRQoL, includes thirty-six items for evaluating physical functioning, role limitations due to physical problems, bodily pain, general health, vitality, social functioning, role limitations due to emotional problems and mental health. Each SF-36 subscale was converted to a scale from 0 to 100; the higher score, the better the HRQoL. A translated version of the SF-36 has been validated in Chinese populations [10].

The HHS ranges from 1 to 100 points, and its domains include pain function (1 item), physical function (7 items), deformity (5 items), and range of motion (5 items) [5]. Pain and physical functions are the two basic considerations and are weighted most heavily in the HHS calculation (44 and 46 points, respectively). Physical functions are classified as daily life activities (3 items, 13 points) and gait (4 items, 33 points). Deformity and range of motion are seldom of primary importance and thus each received 5 points. The higher the score, the better the HRQoL implies.

### Statistical Analysis

The unit of analysis was the individual patient. To compare SF-36 and HHS subscales, raw scores were transformed and scaled from 0 to 100, with higher scores correlating with improved HRQoL.

The generalized estimating equation (GEE) approach is similar to that of repeated measure ANOVA but is more powerful because it can accommodate incomplete data for individual subjects at one or more assessment points without compromising the remaining data for the subject. This approach is also advocated for analyzing incomplete data in longitudinal studies with continuous outcomes [11,26] J. Twisk, Applied longitudinal data analysis for epidemiology, Cambridge University Press, Cambridge (2003). The GEE approach was employed to compare longitudinal changes in SF-36 and HHS subscales before and six months after revision THA. Each HRQoL subscale was used as a dependent variable as a function of time and covariates: age, gender, number of comorbidities, average length of stay and re-hospitalization in 30 days. Variables were entered into the GEE analysis as covariates because they were statistically significant in the univariate analysis and have proven to be consistent predictors of HRQoL in many previous studies [3-5].

Responsiveness estimates were evaluated in terms of percentage of change (PC), effect size (ES) [6-8], standardized response mean (SRM) [6-8] and relative efficiency (RE) [12]. The PC was presented as the mean change scores divided by the baseline scores. The ES was calculated by dividing mean change score by the standard deviation of baseline scores. The SRM was calculated as the mean change score divided by the standard deviation of changed scores. Relative efficiency (RE) is defined as the ratio of the square of the *t*-statistic of the comparator instrument (here, each SF-36 subscale score) over the square of the *t*-statistic of the reference instrument (here, HHS total score). An RE score of 1.0 indicates that the SF-36 is as efficient as the HHS in detecting differences in external indicators of health status. If RE exceeds 1.0, the SF-36 is more efficient than the HHS at detecting differences in external indicators of health status. If RE is lower than 1.0, the SF-36 is less efficient than the HHS. It has been suggested that a one-half standard deviation (SD) change of the mean difference in scores may approximate an MID for some patient-reported outcome instruments, and that evidence from previous studies, physiologic arguments, and statistical theory shows a tendency to converge to the one-half-SD criteria as being meaningful to patients [8,13]. An MCID value was determined by multiplying the SD of the mean difference in scores by 0.5.

Repeated assessment of a single patient can cause complications due to highly correlated observations within the same patient. To address these issues, the bias-corrected and accelerated bootstrap method with 2,000 replications and the modified Jacknife test were used to compare responsiveness estimates between two HRQoL instruments [9,14]. Bootstrapping is a technique for re-sampling numerous random samples drawn from the original sample with replacement [14]. Within each of these samples, calculating the parameter of interest yields an empirical sampling distribution of the estimator of interest from which, without parametric assumptions, probability statements and confidence intervals can be derived. Differences in ES and SRM between the HHS and the SF-36 were estimated, and the bootstrapping method was used to obtain 95% confidence intervals for these differences. The modified Jacknife method is a linear regression between the difference in ES or SRM between two comparable scores (e.g., between SF-36 bodily pain and HHS pain function) as the dependent variable and the centered ES/SRM of one of the two scales (either scale is appropriate) as the independent variable [9]. A regression intercept (value of the SRM/ES difference at which the centered ES/SRM equals zero) larger or smaller than zero with significance p < 0.05 indicates that the two scales significantly differ in responsiveness.

All statistical analyses were performed using Stata Statistical Package, Version 9.0 (Stata Corp, College Station, TX). A p value < 0.05 was considered statistically significant.

## Results

The study sample included twenty-nine (43%) female and thirty-eight (57%) males with a mean age of 70.2 years (standard deviation, 13.1 years; range, 50-92 years). Preoperatively, each patient exhibited an average of 0.6 co-morbidities, and the average length of stay was 6.4 days (standard deviation, 1.7 days). The subjects who remained in the study and those who were lost to follow-up did not significantly differ in baseline age, gender, number of co-morbidities, 30-day re-hospitalization, SF-36 subscale scores, or HHS subscale scores. Therefore, subjects with incomplete information during the study period were assumed to have no significant confounding effects on the statistical results (data not shown). The sample size in this study was sufficient to detect a ten-point difference over time in all SF-36 and HHS subscales, assuming an α of 0.05, a power of 80%, an inter-temporal, between-score correlation of 0.70 and standard deviation of 10 [4,10]. This study was approved by the Institutional Review Board of Kaohsiung Medical University Hospital and Kaohsiung Veterans General Hospital in Taiwan.

Longitudinal changes in all SF-36 and HHS subscales revealed statistically significant improvement (P < 0.05) after adjustment for baseline age, gender, education, number of co-morbidities, operation time, average lengths of stay and re-hospitalization in 30 days (Table 1). The SF-36 and the HHS before and 6 months after revision THA revealed improvement rates of from 12.7% to 52.8% and from 100.0% to 180.0%, respectively. Further, the GEE approach produced the highest mean scores for HHS deformity and range of motion subscales 6 months after revision THA. Specifically, as compared to a relatively low score of 29.6 before revision THA, the mean SF-36 score for role limitations due to physical problems was 41.3 after revision THA, an improvement of 39.8%. The mean SF-36 score for role limitations due to emotional problems changed from 59.0 to 90.2, indicating the role limitations due to emotional problems was the most improved subscale, with an improvement rate of 52.8%. The least improved SF-36 subscale was physical functioning and bodily pain, with an improvement rate of 12.7% and 12.5%, respectively.

**Table 1:** Estimated responsiveness and minimal important differences (MID) for the SF-36 and the Harris Hip Score (HHS)^§^

					Indicators of responsiveness				MID
	Before revision THA	After revision THA	Differences						
Subscale	Mean (S.D.)	Mean (S.D.)	Mean (S.D.)	P value	PC (%)	ES	SRM	RE	
SF-36									
PF	64.1(6.7)	72.2(6.4)	8.1(6.5)	<0.001	12.67	1.22	1.25	0.07	3.25
RP	29.6(14.3)	41.3(7.2)	11.8(9.6)	<0.001	39.77	0.82	1.23	0.10	4.78
RE	59.0(36.2)	90.2(43.7)	31.2(40.0)	<0.001	52.80	0.86	0.78	0.33	19.98
SF	69.8(45.9)	88.2(47.2)	18.4(31.7)	<0.001	26.30	0.40	0.58	0.21	15.83
BP	73.7(22.5)	82.9(24.5)	9.2(29.8)	<0.001	12.54	0.41	0.31	0.09	14.91
VT	53.4(34.0)	76.2(36.0)	22.8(45.6)	<0.001	42.71	0.67	0.50	0.21	22.81
MH	69.4(21.4)	82.2(22.3)	12.9(24.7)	<0.001	18.54	0.60	0.52	0.11	12.37
GH	64.1(42.3)	78.5(48.2)	14.4(28.2)	<0.001	22.46	0.34	0.51	0.12	14.12
HHS									
Pain	34.1(10.4)	95.4(15.7)	61.4(6.9)	<0.001	179.99	5.91	8.92	0.57	3.44
Function	35.9(7.1)	89.1(9.4)	53.3(4.6)	<0.001	148.48	7.49	11.67	0.49	2.28
Deformity	50.0(11.7)	100.0(20.0)	50.0(22.5)	<0.001	100.00	4.28	2.22	0.67	11.26
Motion	50.0(10.1)	100.0(14.7)	50.0(35.7)	<0.001	100.00	4.95	1.40	0.72	17.86
Total scores	36.5(6.6)	93.0(17.8)	56.5(4.9)	<0.001	154.79	8.57	11.58	1.00	2.44

^§^PC = percentage of change; ES = effect size; SRM = standard responsiveness means; RE = relative efficiency; CL = 95% confidence interval at lower limit; CU = 95% confidence interval at upper limit; PF = physical functioning; RP = role limitations due to physical problems; RE = role limitations due to emotional problems; SF = social functioning; BP = bodily pain; VT = vitality; MH = mental health; GH = general health; Pain = pain function; Function = physical function; Deformity = deformity; Motion = range of motion.

The MID in the HHS pain function, physical function, deformity, and total scores (range from 2.28 to 11.26) are generally higher than those of the SF-36 subscales (range from 12.37 to 22.81), except physical functioning and role limitations due to physical problems, during the study period (Table 1). Therefore, the correlation between the HHS and the SF-36 required use of the bootstrap and the modified Jacknife methods to analyze differences in responsiveness.

Because the HHS subscale deformity and range of motion cannot be compared with any SF-36 subscale, we choose physical function and pain function for responsiveness differences comparison (Table 2). The difference may be considered statistically significant at the 0.05 significance level if the confidence interval excludes zero. The HHS revealed significant increases in the ES and SRM of physical function and pain function between the preoperative and 6-month surveys [ES of difference in physical function 6.27 (95% CI: 5.84 to 6.69) and SRM of difference in physical function 10.42 (95% CI: 9.92 to 10.92); ES of difference in pain function 5.50 (95% CI: 5.05 to 5.95) and SRM of difference in pain function 8.61 (95% CI: 8.21 to 9.00)]. Overall, the HHS was statistically more responsive than the SF-36 was in terms of physical function and pain function measurements (p < 0.001).

**Table 2:** Comparative responsiveness estimates of effect size (ES) and standardized response mean (SRM) of the SF-36 and the Harris Hip Score (HHS)

	HHS - SF-36			
	
	**ES (Estimate [95% CI])**^**§**^	**SRM (Estimate [95% CI])**^**§**^	p(ES)*	p(SRM)*
Physical function	6.27 (5.84, 6.69)	10.42 (9.92, 10.92)	<0.001	<0.001
Pain function	5.50 (5.05, 5.95)	8.61 (8.21, 9.00)	<0.001	<0.001

^§^Differences are presented in effect size and standardized response means (95% confidence interval obtained by bootstrapping).

*p: Type I error of the modified Jacknife test comparing two ES or two SRM.

## Discussion

Based on the assessments of the HHS and the SF-36, this comparative study yielded systematic and comprehensive data regarding responsiveness and MID in patients undergoing revision THA.

Analysis of longitudinal changes indicated the role limitations due to physical and emotional problems of the SF-36 exhibited the highest improvement rate. Before surgery, the mean scores for physical and emotional roles were relatively lower than those for any other scale, probably because these roles were limited by the physical and emotional function of patients. The patients could resume their role limitations immediately after revision THA. Consequently, improved role limitations might improve vitality, social functions, general health, mental health and as well as overall quality of life. However, the areas of pain relief and physical function revealed relatively poorer improvement than other functions. This might implicate there was a trend for patients who had had more severe functional problems before the surgery to have poorer pain and physical functions after revision THA [15]. Nevertheless, the items for the role subscales have 5 possible answer levels in version 2 of the SF-36 instead of 2 (present/absent) in version 1. The role subscales are measured more fine graded and more differentiated. The range of possible scores has increased and differences (baseline to follow-up) can be measured more precisely by version 2 [16].

This study is the first to compare the HHS and the SF-36 for responsiveness and MID in revision THA patients treated at two medical centers. The data derived by this study can help clinicians and health researchers decide which measure is most effective for evaluating HRQoL before and after revision THA. The responsiveness estimates for the HHS generally exceeded 0.5, which can be interpreted as medium change [17,18]. Partial subscales of the SF-36 also presented good results in responsiveness estimates and MID, which revealed improvement after surgery. This study also revealed the close algebraic relationship and conceptual differences between ES and SRM estimates, which is consistent with an earlier report by Zou [19].

Schmitt and Fabio [20] contrasted the use of responsiveness indicators at the group level versus the individual patient level. While several other studies in orthopedic surgery and medicine have used MID to compare HRQoL instruments [4,7], no investigators have applied MID calculations to the HHS.

Importantly, although the improvements were in different subscales of the HHS and the SF-36, the estimated responsiveness of the HHS generally was greater than that of the SF-36. However, such the responsiveness estimates in previous studies [3,15] were made using a small sample size or lacked comparative statistical data before and after interventions. Thus, the bootstrap method employed in this study generated a 95% confidence interval. Although the two measures significantly differed in responsiveness, each exhibited superior responsiveness in different subscales. The HHS exhibited superior responsiveness in physical function and pain function subscales.

An acknowledged limitation of this study is the small sample size, which restricts the extent to which the findings can be generalized to larger populations. Future studies are needed to examine outcomes, patient attributes, hospital attributes, care quality, preoperative functional status and related factors in a larger population. Further, the patient outcome may be highly dependent on variables such as operator proficiency, advancing technology and available facilities [21]. However, all procedures evaluated in this study were performed by surgeons with the most experience in revision THA procedures in each of two different institutions, and the potential confounding factors in both responsiveness and MID were controlled simultaneously. Given this design, the surgical outcomes in this study were more representative than those of a single-surgeon study.

To confirm the data regarding the responsiveness and MID of the HHS and the SF-36 scores, Table 3 presents an international data comparison. The findings of this study were compared with those similar studies of United States and European populations [7,22-26]. These studies were selected because they were similar to the current study in terms of sample size, mean age of the population, measurement time points (including pre-operation and at least 6 months postoperative), and, most importantly, the use of both disease-specific and generic measures. The current finding of greater responsiveness of the disease-specific measure in comparison with the generic measure was consistent with all comparable studies examined. Specifically, the increased responsiveness of the disease-specific measure suggests that physical and related functions improve more rapidly and more completely than overall quality of life in patients who undergo revision THA.

**Table 3:** Comparative responsiveness and minimal important differences (MID) of health-related quality of life (HRQoL) instruments reported in previous studies

Authors	Country	No. of subjects	Measurement time intervals	Instrument*	Findings
Shi HY, *et al. *(present study)	Taiwan	67 revision THA	Preoperative and 6-month surveys	HHS, SF-36	HHS revealed greater overall responsiveness than the SF-36 between pre-op and 6-month surveys.

Soohoo NF, *et al. *(2007)[7]	U.S.	89 primary THA	Preoperative and 5 to 17-month surveys	WOMAC, SF-36	The standardized response means (SRM) for the WOMAC ranged from -0.93 to -1.49, and the effect size (ES) ranged from -1.02 to -1.53. The SRM for the SF-36 ranged from 0.22 to 1.64, and the ES ranged from 0.20 to 1.97.

Quintana JM, *et al. *(2005) [22]	Spain	310 primary THA	Preoperative, 6- and 24-month surveys	WOMAC, SF-36	WOMAC exhibited treatment responsiveness superior to the SF-36. The percentage of minimal detectable change (MDC) was higher than 80% for all WOMAC domains, except stiffness (60%), while it was higher than 40% in the physical domains of the SF-36 (physical function, physical role, or bodily pain).

Angst F, *et al. *(2001)[23]	Switzerland	433 hip or knee OA	Preoperative and 3-month surveys	WOMAC, SF-36	SRM = 0.723 for WOMAC and SRM = 0.528 for SF-36 at the end of rehabilitation; SRM = 0.377 for WOMAC and SRM = 0.468 for SF-36 at the three month follow up. In the measurement of function, the WOMAC was significantly more responsive than the SF-36 (SRMs, end of rehabilitation: 0.628 vs. 0.249; three month follow up: 0.235 vs. -0.001).

Hoeksma HL, *et al. *(2003) [24]	Netherlands	75 hip OA	Preoperative and 5-week surveys	HHS, SF-36	The responsiveness ratio for the HHS was high (1.70) compared with walking speed (0.45), pain during walking (0.66), and the subscales of the SF-36-"bodily pain" (0.42) and "physical functioning" (0.36).

Weigl M, *et al. *(2006)[25]	Germany	439 low (upper) back pain and conditions of the lower (upper) extremities patients	Preoperative and 2~4-week surveys	SCQ SF-36 NASS DASH	The condition-specific instruments demonstrated a good responsiveness with an ES ranging between 0.28 and 0.55 and with a SRM between 0.32 and 0.94. The responsiveness of the SF-36 Physical Function scale showed a lower responsiveness than the condition-specific scales.

Lübbeke A, *et al. *(2007) [26]	Switzerland	435 primary THA, 116 revision THA	Preoperative and 5-year surveys	HHS, WOMAC, SF-12	HHS and WOMAC were relatively more responsive for physical and pain functions than the SF-12.

*HHS = Harris Hip Score, SF-36 = Medical Outcomes Study Short Form-36 Health Survey, WOMAC = Western Ontario and McMaster Universities Osteoarthritis Index, SCQ = Self-administered Comorbidity Questionnaire, NASS = North American Spine Society Questionnaire, DASH = Disabilities of Arm, Shoulder, and Hand Questionnaire

## Conclusion

The comparative results of this prospective observational study provide comprehensive and systematic information regarding the expected responsiveness and MID in patients undergoing revision THA. The HHS exhibited responsiveness superior to that of the SF-36 between the preoperative and 6-month surveys. Therefore, clinicians and health researchers may consider weighting the HHS more heavily than the SF-36 to determine treatment effectiveness. Further study may also examine the extent to which the HRQoL instrument is applicable to other forms of orthopaedic surgery.

## Competing interests

The authors declare that they have no competing interests.

## Authors' contributions

HY involved in conception and design, obtaining grants, development of interview guide, acquisition, performed and transcribed the interviews, preparation of data, analyses and interpretation of data, drafting the article. JK, CY, JW, YK, and HC involved in conception and design, analyses and interpretation of data, substantial contribution in revising the article for important intellectual content. KT involved in conception and design, development of interview guide, analyses and interpretation of data, substantial contribution in revising the article for important intellectual content. All authors have read and approved the final manuscript.

## Pre-publication history

The pre-publication history for this paper can be accessed here:

http://www.biomedcentral.com/1471-2474/11/261/prepub
